# Alcohol Use and Abusive or Neglectful Behaviors Among Family Caregivers of Patients With Dementia

**DOI:** 10.1001/jamanetworkopen.2025.6211

**Published:** 2025-04-22

**Authors:** Jessica Andrea Hernandez Chilatra, Wesley Browning, Mustafa Yildiz, Maria Yefimova, Christopher D. Maxwell, Tami Sullivan, Carolyn E. Z. Pickering

**Affiliations:** 1 Department of Research, Cizik School of Nursing, The University of Texas Health Science Center at Houston; 2 McWilliams School of Biomedical Informatics, The University of Texas Health Science Center at Houston; 3 Center for Nursing Excellence & Innovation, University of California, San Francisco; 4 School of Criminal Justice, Michigan State University, East Lansing; 5 Division of Prevention and Community Research, Department of Psychiatry, Yale School of Medicine, New Haven, Connecticut

## Abstract

**Importance:**

The increasing prevalence of dementia presents challenges for family caregivers. Understanding how alcohol use among caregivers relates to abusive and neglectful behaviors (ANBs) toward care recipients with dementia may inform interventions to improve the well-being of both.

**Objective:**

To explore alcohol use patterns among family caregivers of relatives with dementia and investigate whether hazardous drinking and drinking on a given day are independently associated with increased odds of ANBs toward care recipients.

**Design, Setting, and Participants:**

This microlongitudinal cohort study included a baseline survey and 21 days of daily diary surveys among a convenience sample of family caregivers aged 18 years or older who coresided with and provided unpaid care to a community-dwelling relative with dementia across the US. Enrollment was from October 2019 to February 2023.

**Exposures:**

Hazardous drinking at baseline, identified by the Alcohol Use Disorder Identification Test–Consumption screening tool, and daily alcohol consumption recorded in daily diary surveys.

**Main Outcomes and Measures:**

Daily reports assessed engagement in physically aggressive, psychologically aggressive, and/or neglectful behaviors toward care recipients, categorized as binary outcomes. A generalized linear mixed model examined associations between caregivers’ baseline hazardous drinking status and daily alcohol use and odds of ANBs.

**Results:**

Of 453 family caregivers (394 of 451 [87.4%] female; mean [SD] age, 51.6 [14.0] years), 82 (18.1%) screened positive for hazardous drinking. Over the 21-day period, 38 (8.4%) consumed alcohol at least once and 341 (75.3%) engaged in abusive or neglectful behavior at least once. Baseline hazardous drinking was associated with higher odds of engaging in neglectful behaviors (odds ratio [OR], 2.89; 95% CI, 1.74-4.80; *P* < .001) and psychologically aggressive behaviors (OR, 2.32; 95% CI, 1.28-4.19; *P* = .006). Any alcohol use on a given day was associated with greater odds of physically aggressive (OR, 2.32; 95% CI, 1.10-4.88; *P* = .03) and neglectful (OR, 1.66; 95% CI, 1.31-2.10; *P* < .001) behaviors on the same day. No statistically significant interaction between baseline hazardous drinking and daily alcohol use was observed.

**Conclusions and Relevance:**

In this microlongitudinal cohort study of family caregivers of relatives with dementia, hazardous drinking and daily alcohol consumption were significantly and independently associated with increased odds of ANBs, highlighting the need for personalized interventions that address caregiving complexities and alcohol use patterns.

## Key Points

**Question:**

Is hazardous drinking or daily alcohol consumption associated with increased odds of abusive or neglectful behaviors among family caregivers of patients with dementia?

**Findings:**

In this cohort study of 453 family caregivers, screening positive for hazardous drinking was associated with higher odds of neglectful and psychologically aggressive behaviors toward care recipients. Alcohol consumption on a given day, regardless of quantity, was independently associated with higher odds of physically aggressive and neglectful behaviors on the same day.

**Meaning:**

These findings highlight the need for targeted interventions for family caregivers of patients with dementia.

## Teaser

This cohort study investigates whether alcohol consumption among family caregivers of relatives with dementia is associated with abusive and neglectful behaviors toward care recipients.

## Introduction

Hazardous drinking, characterized by excessive alcohol use, is a crucial factor in developing alcohol use disorder (AUD).^1^ It includes binge drinking (≥5 drinks for men or ≥4 drinks for women in 1 sitting) and heavy drinking (≥15 drinks/wk for men or ≥8 drinks/wk for women).^2^ Family caregivers of people with dementia face significant stress and difficulties in managing their emotions, which may increase their risk of hazardous drinking.^3,4^ This is a significant concern since alcohol consumption has been shown to be associated with negative caregiving outcomes, including elder abuse and neglect.^5,6,7^ A recent study on caregivers of people with dementia found that 18% screened positive for hazardous drinking, exceeding general population rates reported by the National Institute on Alcohol Abuse and Alcoholism.^8^

Elder abuse and neglect are growing public health concerns that impact a considerable population at both national and international levels.^9^ The Centers for Disease Control and Prevention defines these as acts or omissions that cause harm to an older person. They include physical aggression, psychological aggression, and neglectful behaviors.^10^ While the worldwide prevalence of abuse and neglect among community-dwelling older adults is about 15%,^11^ it is higher among people with dementia, with over half of family caregivers engaging in physically and psychologically abusive and neglectful behaviors (ANBs).^12,13^

Prior work reported an association between caregiver substance use and increased likelihood of multiple types of abusive behaviors except for neglect.^14^ However, many studies have relied on case investigations or secondary data sources rather than direct self-reports from caregivers, which may underestimate abuse.^15^ Others examined alcohol use in caregivers of people with dementia but used cross-sectional designs that did not capture daily fluctuations in drinking or caregiving behaviors.^16,17^ Additionally, studies examining coping mechanisms and alcohol use among caregivers did not assess how daily drinking patterns relate to caregiving behaviors, making it difficult to establish dynamic associations.^17^

This study was designed to improve on prior research by using a microlongitudinal daily diary design, which allowed for real-time assessment of alcohol use and caregiving behaviors. By collecting data daily over 21 days, this approach overcame the limitations of recall bias to provide a more nuanced understanding of alcohol-related risks in dementia caregiving. We aimed to describe alcohol use patterns in a diverse sample of family caregivers of people with dementia and the association of alcohol use with daily ANBs. The first hypothesis was that caregivers who screened positive for hazardous drinking at baseline would have higher odds of engaging in physically aggressive, psychologically aggressive, or neglectful behaviors compared with caregivers who did not screen positive. The second hypothesis was that on days when alcohol was consumed, caregivers would have greater odds of exhibiting physically aggressive, psychologically aggressive, or neglectful behaviors on the same day.

## Methods

### Participants

This microlongitudinal cohort study included family caregivers of community-dwelling people with dementia across the US who were recruited through print, social media, and community outreach. Eligible participants were caregivers aged 18 years or older who provided unpaid care to and coresided with a relative (spouse or partner, parent or grandparent, or in-law) aged 60 years or older who had mild cognitive impairment or dementia, as identified using the 8-Item Interview to Differentiate Aging and Dementia.^18^ Participants needed to support their relative with at least 1 activity of daily living or 2 instrumental activities of daily living^19^ and have reliable internet access and fluency in English or Spanish. The study was deemed exempt by the institutional review board at the University of Alabama at Birmingham because data were anonymized by a broker. Under the exemption criteria, no written or signed consent was required; however, all participants provided consent online before participation. The study followed the Strengthening the Reporting of Observational Studies in Epidemiology (STROBE) guideline for quality and transparency.^20^

### Procedure

Eligibility was assessed through a multistep process to ensure participant authenticity in online research.^21,22,23^ Participants resided across the US and received a unique link via email and text message reminders. They completed a baseline survey on demographics, mental health, social and emotional well-being, and drinking history. The baseline survey was followed by a series of brief daily diaries for 21 consecutive days about the previous day’s caregiving experiences. These were available online or via interactive voice-response telephone calls. Diaries were available online via any internet-enabled device (eg, smartphones, computers, or tablets) or via interactive voice response between 7 am and 11 am, with hourly email and text reminders during the response window or until completion (availability during the time frame was confirmed during eligibility screening). Participants received a $40 stipend for the baseline survey and $2 per completed daily diary. Enrollment spanned from October 2019 to February 2023.

### Measures

#### Physically Aggressive, Psychologically Aggressive, and Neglectful Behaviors as Outcomes

Daily diaries assessed abuse and/or neglect within the past 24 hours (7 am to 7 am). Three items assessed physical aggression, and 2 assessed psychological aggression, all adapted for daily use from the Conflict Tactics Scale–Revised (CTS-R)^24^ and its modification for older adults (CTS-OA),^25^ consistent with prior research.^12^ Four items assessed neglect—specifically, the omission of necessary care or supervision—using items consistent with established research modified to assess day-level events,^12,26^ including 1 item from the Conflict Tactics Scale for Child Mistreatment.^24^ Responses were coded as binary indicators of physically aggressive, psychologically aggressive, or neglectful behaviors (Table 1).

**Table 1.** Survey Items for Physically Aggressive, Psychologically Aggressive, and Neglectful Behaviors and Alcohol Use

Outcome or variable	Source	Survey
**Physically aggressive behaviors**		
From 7 am yesterday to 7 am this morning:		
Did you pinch, push, shove, or grab your relative; twist their arm or hair; or throw something at them that could have injured them?	CTS-R	Daily diary
Did you bite, hit, kick, punch, choke, or burn your relative?	CTS-R	Daily diary
Did you bruise, scratch, or otherwise physically injure your relative in any way?	CTS-R	Daily diary
**Psychologically aggressive behaviors**		
From 7 am yesterday to 7 am this morning:		
Did you curse, yell, shout at, or speak to your relative in a way you know is not fair or appropriate?	CTS-R	Daily diary
Did you threaten to abandon or put your relative in a nursing home?	CTS-OA	Daily diary
**Neglectful behaviors**		
From 7 am yesterday to 7 am this morning:		
Did you skip care or not help with their (the relative’s) personal hygiene or going to the bathroom even though your relative needed help, such as brushing teeth, bathing, or doing laundry?	Burnes et al,^26^ 2015; Pickering et al,^12^ 2020	Daily diary
Did you skip care or not help at a mealtime even though your relative needed help, such as cooking the food or helping your relative use a fork?	Burnes et al,^26^ 2015; Pickering et al,^12^ 2020	Daily diary
Did you ignore reasonable requests for help from your relative?	Burnes et al,^26^ 2015; Pickering et al,^12^ 2020	Daily diary
Did you leave your relative alone for any period of time even though you thought someone should be there to supervise or help them?	CTS-PC	Daily diary
**Hazardous drinking at baseline**		
In the past 6 mo:		
How often did you have a drink containing alcohol?	AUDIT-C	Baseline
How many drinks did you have on a typical day when you were drinking?	AUDIT-C	Baseline
How often did you have 6 or more drinks on 1 occasion?	AUDIT-C	Baseline
**Alcohol use on a given day**		
From 7 am yesterday to 7 am this morning, did you drink wine, beer or liquor?	Developed for this study	Daily diary

Abbreviations: AUDIT-C, Alcohol Use Disorder Identification Test–Consumption^27^; CTS-OA, Conflict Tactics Scale–Older Adults^12,25^; CTS-PC, Parent-Child Conflict Tactics Scale^24^; CTS-R, Conflict Tactics Scale–Revised.^24^

#### Hazardous Drinking at Baseline and Day-Level Alcohol Use as Variables

The Alcohol Use Disorder Identification Test–Consumption (AUDIT-C) is a brief screening tool aligned with the *Diagnostic and Statistical Manual of Mental Disorders* (Fifth Edition) that is designed to identify individuals at risk for hazardous drinking and active AUD.^27^ The AUDIT-C score ranges from 0 to 12, with a cumulative score of 4 or higher indicating a positive screening result for hazardous drinking in men and a score of 3 or higher indicating the same for women; higher scores indicate increased health and safety risks. We operationalized hazardous drinking at baseline as a binary variable, by which scores meeting or exceeding the sex-specific thresholds were coded as positive and below the thresholds as negative.

To assess daily alcohol consumption, caregivers were asked whether they had consumed alcohol (wine, beer, or liquor) in the past 24 hours (7 am to 7 am) regardless of quantity. The responses represented a binary outcome for any amount of alcohol consumption within 24 hours or none at all.

#### Covariates

Race and ethnicity, poverty status, work hours, and caregiving hours were included as covariates to control for potential confounding, as they are associated with social determinants of abuse or neglect and alcohol use.^28,29^ To avoid stigmatization, race and ethnicity were included as a control variable rather than analyzed as a variable of interest. Race and ethnicity were self-reported using US Census Bureau classifications. Race categories included Asian, Black or African American, Native American or Alaska Native, Native Hawaiian or Other Pacific Islander, and White. Ethnicity was categorized separately as Hispanic, Latino, or of Spanish origin and not Hispanic, Latino, or of Spanish origin. Participants identifying with multiple racial categories were classified as multiracial. For analysis, race and ethnicity categories were dichotomized as non-Hispanic White compared with each minoritized racial or ethnic group. This approach was chosen to adjust for potential confounding rather than to examine racial and ethnic disparities in ANBs. Non-Hispanic White classification applied to individuals identifying as White without additional identification as Black or African American, Asian or Pacific Islander, Hispanic or Latino, Native American or Alaska Native, or multiracial. Income was compared against the 2021 federal poverty limit to classify whether participants’ socioeconomic status was above or below the poverty level.^30^

Caregiving responsibilities were measured by assessing hours spent caring for additional family or friends with a health problem. Since all participants were required to provide unpaid care, coreside with the person with dementia, and assist with at least 1 activity of daily living or 2 instrumental activities of daily living, caregiving intensity for the people with dementia was considered a constant and was not included as a variable. Future research should explore variations in caregiving intensity and their impact on ANBs.

### Statistical Analyses

Missing data were assumed to be missing at random due to the low percentage of missingness at the item level and were imputed in a 2-step process using multiple imputation for all nondemographic baseline variables and single imputation for diary-level missingness. First, baseline-level data were multiply imputed (m = 40) using the mice package in R, version 4.3.1 (R Project for Statistical Computing).^31^ These imputations incorporated available cognitive and behavioral variables and ensured internal consistency across datasets. Demographic variables were not imputed to avoid introducing bias, as missingness in these variables may not be at random. Second, diary-level missingness was imputed in Mplus, version 8.8 (Muthén & Muthén) using a model-free single imputation approach (m = 1 per imputed dataset) incorporating baseline variables, including exposures (alcohol use) and outcomes (ANBs). To reduce computational complexity, a single randomly selected dataset from the 40 imputations was used for analysis rather than pooling results across all imputations. Consistency was assessed by comparing key summary statistics across the 40 datasets, ensuring that estimates of central tendency and variance were stable. Participants with at least 1 completed diary entry were included in the analysis. No formal sample size calculation was conducted; the sample was based on feasibility and recruitment efforts.

A generalized linear mixed model evaluating data from diary days nested within caregivers was used to evaluate associations between drinking behaviors and odds of ANBs. Separate models were run for physically aggressive, psychologically aggressive, and neglectful behaviors, with hazardous drinking at baseline and daily alcohol use as independent variables. Covariates included age, sex, non-Hispanic White race and ethnicity, caregiving hours per week, work hours per week, and household income below the poverty level. Variance inflation factors were examined for all variables, confirming no evidence of multicollinearity (<2 for all covariates), supporting their inclusion.

We also evaluated whether hazardous drinking at the person level and alcohol use at the day level were independently associated with abusive and neglectful behaviors. To correct for multiple comparisons in descriptive analyses, we applied false discovery rate correction using the Benjamini-Hochberg procedure. This adjustment controls the overall type I error rate across demographic and outcome comparisons. Analyses were conducted in R, version 4.3.1, and RStudio, version 2023.03.0.31 (RStudio, PBC).^32^ Two-sided *P* < .05 was considered significant.

## Results

This study included 453 family caregivers (394 of 451 [87.4%] female and 57 of 451 [12.6%] male; mean [SD] age, 51.6 [14.0] years). They completed 7783 diary days across the 21-day period, with 1730 days imputed, maintaining a diary-level missingness rate of 22.22% across 9513 days with less than 1% item-level missingness. Of 371 caregivers with available data, 24 (6.5%) were Asian; 101 (27.2%), Black or African American; 5 (1.3%), Native American or Alaska Native; 0, Native Hawaiian or other Pacific Islander; 232 (62.5%), non-Hispanic White; and 9 (2.4%), multiracial. By ethnicity, 105 of 453 caregivers (23.2%) were Hispanic, Latino, or of Spanish origin, and 348 of 453 (76.8%) were not Hispanic, Latino, or of Spanish origin. Of 453 caregivers, 317 (69.9%) were caring for a parent, in-law, or stepparent, and most had at least some college-level education, including 234 (51.7%) who held a 4-year degree and 148 (32.7%) who had some college or an associate’s degree. Regarding alcohol consumption, 82 caregivers (18.1%) screened positive for hazardous drinking at baseline and 172 (38.0%) reported that they consumed alcohol at least once over the 21-day period. Additionally, 341 caregivers (75.3%) engaged in at least 1 instance of ANB over the 21-day period. Table 2 presents full participant demographics, and the Figure illustrates the proportions of ANBs by hazardous drinking and daily drinking status.

**Table 2.** Descriptive Statistics for Demographic Characteristics and Primary Outcome Measures

Characteristic	Participants^a^			*P* value^b^
	Total	AUDIT-C among caregivers		
		Negative	Positive	
**Caregiver demographics**				
No. (%)	453 (100)	371 (82.3)	82 (17.7)	NA
Age, mean (SD), y	51.6 (14.0)	51.9 (13.6)	49.9 (15.6)	.65^c^
Sex, No./total No. (%)				
Female	394/451 (87.4)	326/369 (88.3)	68/82 (82.9)	.64^d^
Male	57/451 (12.6)	43/369 (11.7)	14/82 (17.1)	NA
Race, No./total No.				
Asian	24/371 (6.5)	19/299 (6.3)	5/72 (6.9)	.86^e^
Black or African American	101/371 (27.2)	81/299 (27.1)	20/72 (27.8)	.86^d^
Native American or Alaska Native	5/371 (1.3)	3/299 (1.0)	2/72 (2.8)	.65^e^
Native Hawaiian or other Pacific Islander	0	NA	NA	NA
Non-Hispanic White	232/371 (62.5)	191/299 (63.9)	41/72 (56.9)	.86^d^
Multiracial	9/371 (2.4)	5/299 (1.7)	4/72 (5.6)	.20^e^
Ethnicity				
Hispanic, Latino, or of Spanish origin	105 (23.2)	81 (21.8)	24 (29.3)	.80^d^
Not Hispanic, Latino, or of Spanish origin	348 (76.8)	290 (78.2)	58 (70.7)	NA
Spouse or partner relationship, No./total No. (%)	101/439 (23.0)	83/358 (23.2)	18/81 (22.2)	.86^d^
Below poverty level, No./total No. (%)	109/427 (25.5)	99/349 (28.4)	10/78 (12.8)	.06^d^
Time per wk worked for pay, mean (SD), h	13.6 (17.1)	12.4 (16.5)	18.6 (18.6)	.08^c^
Time per wk caring for other, mean (SD), h	5.1 (11.6)	4.8 (10.8)	6.3 (14.8)	.65^f^
EAN on ≥1 d during the 21 d				
Neglect	294 (64.9)	229 (61.6)	65 (79.3)	.004^d^
Psychologically aggressive behavior	178 (39.3)	136 (36.7)	42 (51.9)	.04^d^
Physically aggressive behavior	42 (9.3)	32 (8.6)	10 (12.3)	.67^d^
**Care recipient demographics**				
Age, mean (SD), y	78.6 (9.1)	78.6 (9.0)	78.3 (9.4)	.87^f^
Sex, No./total No. (%)				
Female	265/452 (58.6)	221/370 (59.7)	44/82 (53.7)	.86^d^
Male	187/452 (41.4)	149/370 (40.3)	38/82 (46.3)	NA
Race, No./total No. (%)				
Asian	26/382 (6.8)	19/307 (6.2)	7/75 (9.3)	.65^e^
Black or African American	105/382 (27.5)	85/307 (27.7)	20/75 (26.7)	.86^d^
Native American or Alaska Native	8/382 (2.1)	7/307 (2.3)	1/75 (1.3)	>.99^e^
Native Hawaiian or other Pacific Islander	2/382 (0.5)	1/307 (0.3)	1/75 (1.3)	.86^e^
Non-Hispanic White	234/382 (61.3)	190/307 (61.9)	44/75 (58.7)	.86^d^
Multiracial	7/382 (1.8)	5/307 (1.6)	2/75 (2.7)	.86^e^
Ethnicity				
Hispanic, Latino, or of Spanish origin	97 (21.4)	82 (22.1)	15 (18.3)	.80^d^
Not Hispanic, Latino, or of Spanish origin	356 (78.6)	289 (77.9)	67 (81.7)	NA
Military veteran	71 (15.8)	57 (15.5)	14 (17.5)	.86^d^

Abbreviations: AUDIT-C, Alcohol Use Disorder Identification Test; EAN, elder abuse or neglect; NA, not applicable.

^a^ Data are presented as number (percentage) of participants unless otherwise indicated.

^b^ *P* values were adjusted using the false discovery rate correction for multiple comparisons to control the overall type I error rate.

^c^ Welch *t* test.

^d^ χ^2^ test.

^e^ Fisher exact test.

^f^ Student *t* test.

**Figure.**
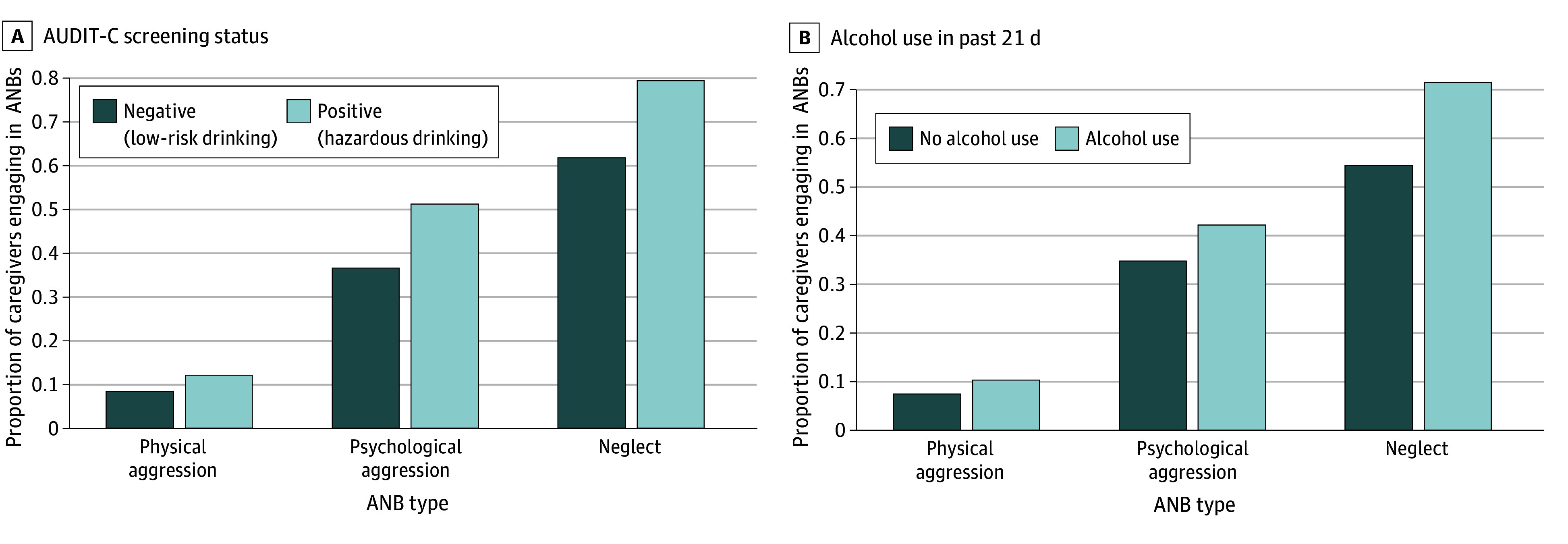
Proportion of Participants Reporting Abusive and Neglectful Behaviors (ANBs) by Hazardous Drinking Status and Daily Alcohol Consumption AUDIT-C indicates Alcohol Use Disorder Identification Test–Consumption.

### Models for Caregivers’ Alcohol Use Patterns

Table 3 shows that screening positive for hazardous drinking at baseline was associated with significantly increased odds of psychologically aggressive (odds ratio [OR], 2.32; 95% CI, 1.28-4.19; *P* = .006) and neglectful (OR, 2.89; 95% CI, 1.74-4.80; *P* < .001) behaviors. The data did not show a significant increase in daily odds of physically aggressive behaviors in caregivers who screened positive for hazardous drinking at baseline (OR, 1.00; 95% CI, 0.07-14.51; *P* > .99). On days when alcohol was consumed, caregivers had greater odds of engaging in physically aggressive behaviors (OR, 2.32; 95% CI, 1.10-4.88; *P* = .03). Similarly, there was an increase in the odds of neglectful behaviors on those days (OR, 1.66; 95% CI, 1.31-2.10; *P* < .001). There was no significant change in the probability of psychologically aggressive behaviors on days when caregivers consumed alcohol (OR, 1.24; 95% CI, 0.88-1.74; *P* = .22). Table 4 shows that the models did not reveal any interactions between hazardous drinking, daily alcohol use, and the daily odds of physically aggressive, psychologically aggressive, or neglectful behaviors.

**Table 3.** Generalized Linear Mixed Model for the Association of Hazardous Drinking and Daily Alcohol Use With Odds of Daily Abusive and Neglectful Behaviors

Caregiver characteristic	Type of abuse					
	Neglect		Psychologically aggressive behavior		Physically aggressive behavior	
	OR (95% CI)	*P* value	OR (95% CI)	*P* value	OR (95% CI)	*P* value
Age^a^	0.99 (0.97-1.00)	.09	1.00 (0.99-1.02)	.61	0.97 (0.89-1.05)	.44
Female sex	0.84 (0.44-1.61)	.61	0.46 (0.22-0.97)	.04	0.10 (0.01-1.28)	.08
Non-Hispanic White^b^	0.35 (0.23-0.54)	<.001	1.44 (0.87-2.38)	.16	0.69 (0.07-6.85)	.75
Above poverty level	0.46 (0.29-0.73)	<.001	0.92 (0.52-1.63)	.77	0.82 (0.06-10.78)	.88
Hours per wk worked for pay^a^	0.99 (0.98-1.01)	.42	0.99 (0.98-1.01)	.28	0.98 (0.91-1.05)	.52
Hours per wk caring for other^a^	0.99 (0.97-1.01)	.24	1.00 (0.98-1.02)	.87	0.99 (0.90-1.09)	.88
Screened positive for hazardous drinking	2.89 (1.74-4.80)	<.001	2.32 (1.28-4.19)	.006	1.00 (0.07-14.51)	>.99
Alcohol use on a given day	1.66 (1.31-2.10)	<.001	1.24 (0.88-1.74)	.22	2.32 (1.10-4.88)	.03

Abbreviation: OR, odds ratio.

^a^ Modeled per unit increase.

^b^ Compared with the minoritized racial and ethnic groups listed in the Methods section.

**Table 4.** Generalized Linear Mixed Model for Interaction Between Hazardous Drinking and Daily Alcohol Use as Factors Associated With Daily Abusive and Neglectful Behaviors

Caregiver characteristic	Type of abuse					
	Neglect		Psychologically aggressive behavior		Physically aggressive behavior	
	OR (95% CI)	*P* value	OR (95% CI)	*P* value	OR (95% CI)^a^	*P* value^a^
Age^b^	0.99 (0.97-1.00)	.10	1.00 (0.99-1.02)	.66	NA	NA
Female sex	0.84 (0.44-1.61)	.61	0.46 (0.22-0.97)	.04	NA	NA
Non-Hispanic White^c^	0.35 (0.23-0.54)	<.001	1.44 (0.87-2.38)	.16	NA	NA
Above poverty level	0.46 (0.29-0.73)	.001	0.92 (0.52-1.62)	.76	NA	NA
Hours per wk worked for pay^b^	0.99 (0.98-1.01)	.42	0.99 (0.98-1.01)	.28	NA	NA
Hours per wk caring for other^b^	0.99 (0.97-1.01)	.24	1.00 (0.98-1.02)	.87	NA	NA
Screened positive for hazardous drinking	3.03 (1.80-5.11)	<.001	2.06 (1.11-3.83)	.02	1.12 (0.07-14.51)	.99
Alcohol use on a given day	2.13 (1.04-4.38)	.04	0.56 (0.17-1.82)	.33	3.18 (0.41-24.50)	.27
Interaction between AUD and daily drinking	0.84 (0.52-1.35)	.47	1.66 (0.81-3.40)	.16	0.82 (0.20-3.33)	.78

Abbreviations: AUD, alcohol use disorder; NA, not applicable; OR, odds ratio.

^a^ The variable for physical aggression was not included in the model.

^b^ Modeled per unit increase.

^c^ Compared with the minoritized racial and ethnic groups listed in the Methods section.

## Discussion

To our knowledge, this is the first study to address the critical need to explore the connection between alcohol use, hazardous drinking behaviors, and the daily risk of family caregivers engaging in abusive or neglectful behaviors toward their relatives with dementia. The findings of our study both support and challenge the initial hypotheses that physically aggressive, psychologically aggressive, or neglectful behaviors on a given day may increase in association with screening positive for hazardous drinking at baseline and any quantity of alcohol use on the same day as the behavior. Different associations were found depending on the type of abuse and neglect.

In line with our first hypothesis, hazardous drinking was independently associated with higher odds of engaging in neglectful and psychologically aggressive behavior. Previous research has shown that hazardous and harmful alcohol consumption by parents is associated with significantly worsened internalizing disorders in children, which are associated with emotional neglect, emotional abuse, and physical and psychological abuse.^33^ Although there has been extensive research on the association between parental alcohol use and child neglect and abuse,^34,35,36^ there is a notable gap in the literature regarding neglect among older adults and the impact of caregiver alcohol use. Contrary to our expectations, our study did not find an increase in physically aggressive behavior among caregivers who screened positive for hazardous drinking at baseline. However, a potential explanation is that hazardous drinking among caregivers is associated with avoidance coping strategies in managing the stress of caring for family members with dementia.^4^ Consequently, these caregivers may be more inclined toward neglectful behaviors as a means of avoidance rather than engaging in physical aggression. Moreover, alcohol-related aggression is not universally observed in all chronic consumers of alcohol or individuals with alcohol dependence.^37^

As for our second hypothesis, we found that caregivers who consumed alcohol on a given day, regardless of quantity or type, exhibited greater odds of engaging in physically aggressive and neglectful behaviors on the same day compared with days with no alcohol consumption. This finding emphasizes the real-time association of daily alcohol consumption with caregiving behaviors. For physical aggression, it is possible that alcohol consumption situationally reduces emotion regulation abilities during experiences of stress and increases the risk of conflict-based tactics.^38^ However, it is also possible that the association is bidirectional, meaning that some caregivers may turn to alcohol after engaging in abusive or neglectful behaviors as a way to cope with feelings of shame, guilt, or stress.^39^ Alternatively, challenging caregiving situations may contribute to both ANBs and later alcohol use. Since our study did not establish temporal order within the 24-hour period, future research should use relevant research designs and methods to better capture the sequence of events. Research should extend beyond the consumption of alcohol to explore the daily contextual circumstances of drinking that may influence the risk of ANBs (eg, drinking alone vs in a social setting). This approach is supported by previous studies showing that alcohol either increased or decreased aggressive behavior depending on individual traits and the situation.^40,41^

The models indicated no significant interaction of hazardous drinking status and daily drinking behaviors with the odds of engaging in abusive or neglectful behaviors toward the care recipient, suggesting that the risks associated with hazardous drinking and the effects of daily alcohol use are independent. Based on our hypothesis, we expected the associations between daily drinking and abuse and neglect to be heightened among caregivers engaging in hazardous drinking. The absence of interaction effects contrasts with prior research suggesting that chronic and acute drinking behaviors might synergistically increase the degree of violence.^42^ These findings suggest that the subset of caregivers experiencing AUD is a high-risk group for use of abuse and neglect who may experience multiple benefits from interventions aimed at managing drinking behaviors. Regular alcohol screening, as recommended by the US Preventive Services Task Force,^43^ should be integrated into caregiver support programs, such as Resources for Enhancing Alzheimer Caregiver Health (REACH),^44^ to prevent poor caregiver outcomes, like the use of ANBs. Incorporating real-time support systems, such as smartphone applications offering immediate access to coping mechanisms and crisis intervention resources, could enhance caregivers’ well-being and reduce hazardous drinking behaviors.^45,46^ The finding that daily alcohol consumption, regardless of type or quantity, was associated with higher odds of ANBs suggests that these are generalizable risk factors that need to be considered in any multicomponent intervention to address abuse and neglect in the dementia caregiving context. Future research should investigate the mechanistic processes by which daily drinking impacts the likelihood of abuse and neglect to inform the development of these multicomponent behavioral interventions.

### Strengths and Limitations

A strength of this study is that it innovatively applied a microlongitudinal design to explore the dynamic association between alcohol use and caregiving behaviors. However, certain limitations should be acknowledged. While the dataset is diverse, findings may not be generalized due to convenience sampling and the lack of comparable representative studies. While race and ethnicity were included as covariates to control for confounding, this approach did not allow for subgroup-specific analyses of racial and ethnic disparities in ANB behaviors. Future research should explore these differences using stratified models. Additionally, social desirability and expectation bias may have influenced self-reporting of alcohol use and caregiving behaviors, as is common in self-reported data. Expectancy biases may also have affected how caregivers reported both alcohol use and ANBs, potentially underestimating or overestimating these behaviors based on personal or societal expectations. Future research should use objective measures, such as biomarkers or passive monitoring, to reduce these biases. This study did not account for census region or geography, and poverty thresholds vary by location and household size. Future research should explore these contextual factors in dementia caregiving. Furthermore, while this study assumed an association between substance use and abusive and neglectful behaviors, it is possible that the association is bidirectional or that substance use occurs as a coping mechanism after ANBs. Family caregivers may experience increased stress the day after using an ANB.^47^ Therefore, future research should consider and investigate these alternative pathways.

In addition, while multiple imputation was used for baseline-level data, single imputation was applied at the diary level for computational feasibility, which may have led to slightly narrower 95% CIs and lower *P* values. Future studies should consider fully implementing multiple imputation across all levels.

## Conclusions

This cohort study found that hazardous drinking and daily alcohol consumption among family caregivers of relatives with dementia were significantly and independently associated with increased odds of ANBs. This study contributes substantially to the existing body of literature in the field of dementia caregiving and alcohol use by providing insights into the association between hazardous drinking patterns, daily alcohol use, and the use of abusive and neglectful behaviors. The findings underscore the need for interventions addressing both baseline hazardous drinking and daily alcohol consumption. The study provides evidence suggesting that potential subgroups within the dementia caregiver population may be prone to abusive and neglectful behaviors—information that is crucial for tailored interventions.

## Notes

Supplement.
Data Sharing Statement
