# Supplementary material for: Alcohol Use and Abusive or Neglectful Behaviors Among Family Caregivers of Patients With Dementia
Source: JAMA Netw Open. 2025 Apr 22;8(4):e256211. doi: 10.1001/jamanetworkopen.2025.6211 (PMC12015665; doi:10.1001/jamanetworkopen.2025.6211)
Supplement: Supplement. — Data Sharing Statement [file jamanetwopen-e256211-s001.pdf]

Data Sharing Statement

Hernandez Chilatra. Alcohol Use and Abusive or Neglectful Behaviors Among Family Caregivers of Patients With Dementia. *JAMA Netw Open*. Published April 22, 2025. doi:10.1001/jamanetworkopen.2025.6211

Data
Data available: Yes
Data types: Deidentified participant data
How to access data: carolyn.e.pickering@uth.tmc.edu
When available: beginning date: 03-01-2025

Supporting Documents
Document types: None

Additional Information
Who can access the data: Data will be made available to researchers whose proposed use of the data has been approved by the principal investigator (via email: carolyn.e.pickering@uth.tmc.edu).
Types of analyses: The data will be made available for any purpose, including exploratory, hypothesis-generating, and confirmatory analyses related to elder abuse, caregiver stress, and health outcomes.
Mechanisms of data availability: The data will be made available through the National Archive for Computerized Data on Aging (NACDA) via ICPSR. Researchers will need to follow the NACDA approval process to gain access to the data.
